# Application effect of lattice laser in facial rejuvenation: A protocol for systematic review and meta-analysis

**DOI:** 10.1097/MD.0000000000021814

**Published:** 2020-08-21

**Authors:** Dan Yan, Zechun Huang, Anli Zhang, Shuaihua Li, Yao Xiao

**Affiliations:** Department of Plastic and Cosmetic Surgery, Chenzhou First People's Hospital, Chenzhou, China.

**Keywords:** acial rejuvenation, lattice laser, meta-analysis, systematic review

## Abstract

**Background:**

Various techniques have been applied in facial rejuvenation and lattice laser is the most accepted. However, the application effect of lattice laser in facial rejuvenation is unclear. This study aims to evaluate the application effect of lattice laser in facial rejuvenation.

**Methods:**

Randomized controlled trials of lattice laser in facial rejuvenation will be searched in PubMed, EMbase, Web of Science, Cochrane Library, China National Knowledge Infrastructure, WanFang, the Chongqing VIP Chinese Science and Technology Periodical Database, and China biomedical literature database from inception to July 2020. And Baidu Scholar, International Clinical Trials Registry Platform, Google Scholar, and Chinese Clinical Trials Registry will be searched to obtain more relevant studies comprehensively. Two researchers will perform data extraction and risk of bias assessment independently. Statistical analysis will be conducted in RevMan 5.3.

**Results:**

This study will sum up the present evidence so far by exploring the application effect of lattice laser in facial rejuvenation.

**Conclusions:**

The findings of the study will provide helpful evidence for the application effect of lattice laser in facial rejuvenation, promoting clinical practice, and further scientific research.

**Ethics and dissemination:**

The private information from individuals will not publish. This systematic review also will not involve endangering participant rights. Ethical approval is not required. The results may be published in a peer-reviewed journal or disseminated in relevant conferences.

**OSF registration number:**

DOI 10.17605/OSF.IO/QF6H5

## 1 Introduction

Aging is a spontaneous and inevitable process of living things over time. It is a complex natural phenomenon characterized by structural degeneration and functional decline, as well as loss of adaptability and resistance.^[1]^ Skin aging is the appearance of aging damage to the skin function, the skin's ability to protect and regulate the body, and other decreased, so that the skin cannot adapt to the changes in the internal and external environment. Skin aging is divided into endogenous and exogenous. Endogenous aging refers to the natural aging of the skin with the growth of age, which is manifested as whitening of the skin, fine wrinkles, decreased elasticity, and sagging of the skin.^[2]^ The main cause of exogenous aging is the light aging caused by the sun, which is manifested as wrinkles, skin relaxation, roughness, pale yellow, or grayish yellow skin discoloration, telangiectasia, pigment spot formation, and so on.^[3]^ For beauty lovers, skin care is a required course. With the development of medical technology, various techniques have been applied to facial rejuvenation, including facial wrinkling surgery, hyaluronic acid injection, botulinum toxin injection, facial fat filling, and so on. The facial contour and lines can be reconstructed by technical means, and the color and elasticity of skin can be restored to make the appearance look younger.^[4]^ However, these methods are traumatic and can cause facial stiffness and other side effects, while laser wrinkle removal has become one of the most commonly used methods due to its simple operation, small trauma, and long duration. It can eliminate all kinds of wrinkles, improve the appearance of eyebrows, and eyes, make the nasolabial groove shallower, obviously improve the appearance of buccal and mandibular margin, and restore the youthful appearance of the face.^[5]^ Due to the difficulty in controlling the depth of skin in exfoliative lasers for facial rejuvenation, exudation, scab, swelling, erythema, pigmentation, and even some adverse effects such as infection and scar healing may occur.^[6]^ In order to improve the shortcoming of exfoliative lasers, non-exfoliative lasers represented by lattice laser has been widely used. Shorter operating time and fewer complications resulted in more pronounced facial rejuvenation.^[7]^

Lattice laser can generate an array of tiny beams of light to act on the skin. After the laser energy is absorbed by the skin tissue water, a number of tiny thermal damage areas of columnar structure are formed, called microscopic treatment zones, which then causes a series of skin biochemical reactions to tighten the skin, tender the skin, and remove the spots. Relevant research suggested that lattice laser could rejuvenate the face and reduce side effects.^[8]^ Therefore, lattice laser in facial rejuvenation is the most accepted. However, there is no systematic review and meta-analysis regarding the application effect of lattice laser in facial rejuvenation. Thus, this study will assess the application effect of lattice laser in facial rejuvenation.

## 2 Methods

### 2.1 Study registration

This protocol of systematic review and meta-analysis has been drafted under the guidance of the preferred reporting items for systematic reviews and meta-analyses protocols (PRISMA-P). Moreover, it has been registered on open science framework (OSF) (registration number: DOI 10.17605/OSF.IO/QF6H5)

### 2.2 Ethics

Ethical approval is not required as there is no patient recruitment and personal information collection, and the data included in our study are from published literature.

### 2.3 Inclusion criteria for study selection

#### 2.3.1 Type of studies

Randomized controlled trials (RCTs) including lattice laser in facial rejuvenation could be included. The language is limited to Chinese and English.

#### 2.3.2 Type of participants

All included patients met the diagnosis of facial skin aging, regardless of nationality, race, age, gender, and source of cases.

#### 2.3.3 Type of interventions

The control group was treated with laser measures, and laser type was not limited but cannot include lattice laser; the treatment group was treated with lattice laser. The duration of treatment in both groups was not limited.

#### 2.3.4 Type of outcome measures

The main outcome measure was clinical efficacy. Facial skin elasticity increases, wrinkles become significantly lighter, wrinkles, and relaxation improved more than 75%, which is significantly effective; the treatment of facial wrinkles and skin elasticity improved by 25% to 75%, which is effective; facial wrinkles, and skin elasticity improved less than 25%, or even no improvement, is invalid.^[9]^

The secondary outcome:

(1) VAS score was used to evaluate the pain degree immediately after treatment. The higher the score, the more severe the pain was;
(2) the changes of smoothness, skin color, wrinkles, and relaxation of facial skin before and after treatment were observed;
(3) after treatment, the satisfaction of patients was investigated. The results were divided into very satisfied, relatively satisfied, general, and dissatisfied. The total satisfaction rate = very satisfactory rate + more satisfactory rate;
(4) the adverse reactions after treatment were observed.

### 2.4 Exclusion criteria

(1) The treatment group used other laser measures;
(2) the outcome indicators of the original study did not meet the requirements;
(3) select literature with the most complete data if there are duplicate published literature;
(4) literature with incorrect or incomplete data that cannot be obtained after contacting the author.

### 2.5 Search strategy

PubMed, EMbase, Web of Science, Cochrane Library, China National Knowledge Infrastructure, the Chongqing VIP Chinese Science, WanFang, Technology Periodical Database, and China biomedical literature database were searched by computer to collect RCTs of lattice laser in facial rejuvenation, and the retrieval time was from the establishment of every database to July 2020. At the same time, search Baidu, International Clinical Trials Registry Platform, Google Scholar, and Chinese Clinical Trials Registry to get more comprehensive data. In addition, the references of the included literatures are traced to supplement and obtain the relevant literatures. Medical Subject Headings and free words are used in the retrieval. Keywords were: “laser” and “facial rejuvenation,”, et al PubMed retrieval strategies are shown in Table 1.

**Table 1:**
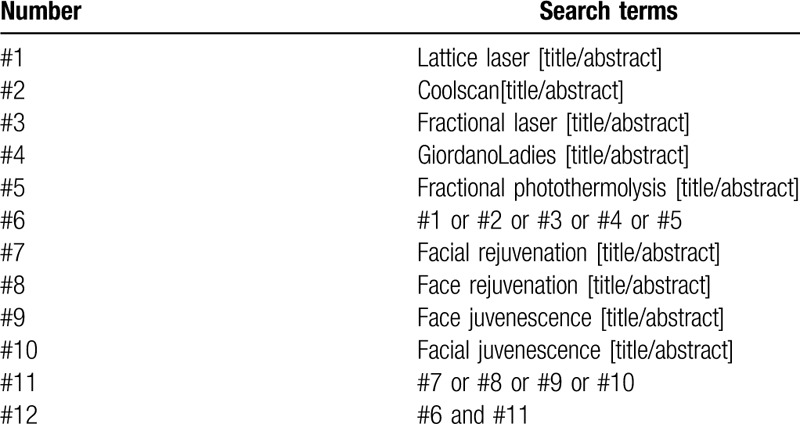
Search strategy in PubMed database.

### 2.6 Data extraction

**Figure 1:**
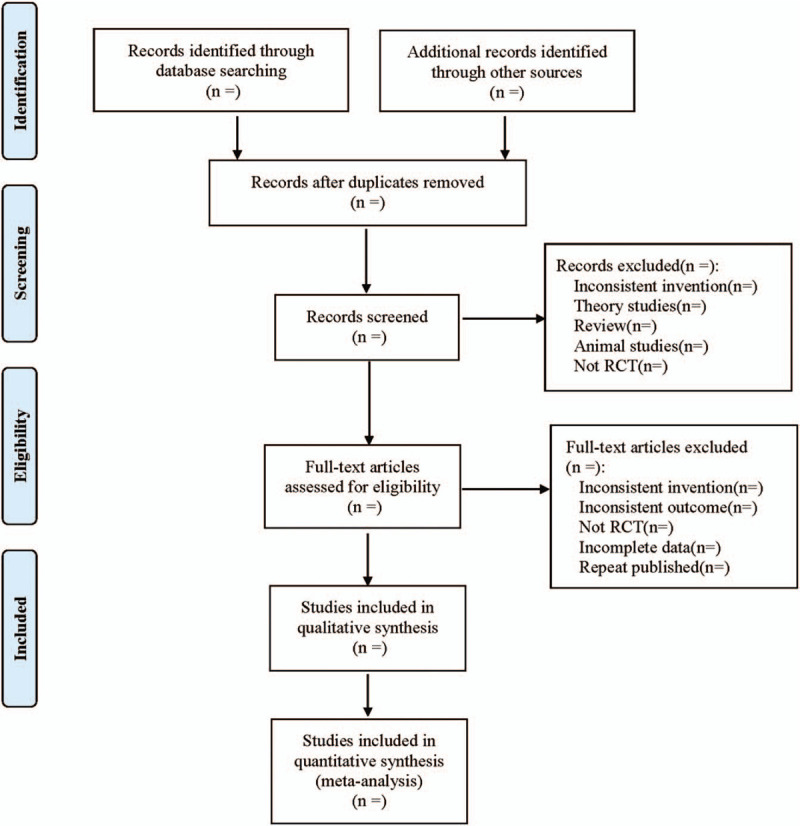
Flow diagram.

Endnote X7 was used for literature management. Two researchers independently screened the literature, extracted the data, and cross checked them. In case of disagreement, the third researcher was consulted to assist in judgment. In the process of literature screening, first read the title and abstract, and then read the full text after excluding the obviously unrelated literature to determine whether it is included. Excel 2019 was used to set up a data extraction table to extract data. The literature screening process is shown in Figure 1.

The extraction contents were as follows:

(1) Included basic research information (study title, first author, publication time, sample size, sex ratio, average age, etc);
(2) information about specific plan (laser used in the treatment group and the control group, its dose, course of treatment, etc);
(3) Risk evaluation items of bias in RCTs;
(4) Main outcome indicators and secondary outcome indicators data, etc. The Newcastle Ottawa scale was used for quality assessment.^[10]^

### 2.7 Risk of bias assessment

Two researchers independently evaluated the risk of bias in RCTs in accordance with the Cochrane Handbook of Systematic Reviewers, including the following items: random sequence generation, allocation concealment, blinding of participants, and personnel, blinding of outcome assessment, incomplete outcome data, selective reporting, and other bias. The quality of studies was classified as being at of high, unclear, or low risk of bias. In case of disagreement, a third researcher decided.

### 2.8 Statistical analysis

#### 2.8.1 Data synthesis

The RevMan 5.3 software (provided by the Cochrane Collaboration) was used for statistical analysis. For dichotomous variables, relative risk was selected as the statistic for the dichotomous variable. For continuous variables, weighted mean difference was selected when the tools and units of measurement indicators are the same, standardized mean difference was selected with different tools or units of measurement, and all the above were represented by effect value and 95% Confidence interval. Heterogeneity test: Q test was used to qualitatively determine inter-study heterogeneity. If *P*≥.1, there was no inter-study heterogeneity, If *P* < .1, it indicated inter-study heterogeneity. At the same time, I^2^ value was used to quantitatively evaluate the inter-study heterogeneity. If I^2^≤50%, the heterogeneity was considered to be good, and the fixed-effect model was adopted. If I^2^ > 50%, it was considered to have significant heterogeneity, the source of heterogeneity would be explored through subgroup analysis or sensitivity analysis. If there was no obvious clinical or methodological heterogeneity, it would be considered as statistical heterogeneity, and the random-effect model would be used for analysis. Descriptive analysis was used if there was significant clinical heterogeneity between the 2 groups and subgroup analysis was not available.

#### 2.8.2 Dealing with missing data

If data is missing or incomplete, we will contact the relevant authors to obtain the data. If not, this study will be removed.

#### 2.8.3 Heterogeneity and subgroup analysis

In order to reduce the clinical heterogeneity between studies, subgroup analysis was conducted according to the age, which were divided into minors, adults, and the elderly. Subgroup analysis was carried out for the included studies according to the course and frequency of lattice laser in facial rejuvenation.

#### 2.8.4 Sensitivity analysis

In order to test the stability of meta-analysis results of indicators, a one-by-one elimination method will be adopted for sensitivity analysis.

#### 2.8.5 Reporting bias

For the major outcome indicators, if the included study was ≥10, funnel plot was used to qualitatively detect publication bias. Egger and Begg test are used to quantitatively assess potential publication bias.

#### 2.8.6 Evidence quality evaluation

The Grading of Recommendations Assessment, Development, and Evaluation (GRADE) will be used to assess the quality of evidence. It contains 5 domains (bias risk, consistency, directness, precision, and publication bias). And the quality of evidence will be rated as high, moderate, low, and very low.

## 3 Discussion

Traditional lasers include stripping laser and non-stripping laser, the stripping laser is represented by CO^2^ laser and erbium-doped yttrium aluminum garnet (YAG) laser, the non-stripping laser is represented by YAG laser and semiconductor laser.^[11]^

The principle of lattice laser technology is to selectively create a 3-dimensional heat loss area in the dermis, resulting in epidermal necrosis, and regeneration. However, the tissue cells outside the heat loss area are not damaged and can be quickly repaired by cell migration.^[12–13]^ So as to achieve the purpose of skin tightening, removing aging skin, and removing skin pigment spots. Generally speaking, the epithelium of the area treated by the lattice laser can be completely recovered within 24 hours, so that the risk of skin infection is low, and the erythema period of the skin is also shortened. Because of its advantages of short recovery time and less adverse reactions, lattice laser is increasingly widely used in the treatment of skin scars and skin pigments.^[14–15]^ The non-stripping lattice laser mainly includes erbium glass laser or erbium-doped YAG laser, etc. Stripping lattice laser can be divided into 3 types: strong stripping, medium stripping, and micro stripping.^[16]^

At present, lattice laser is widely used in the treatment of diseases in plastic surgery, like facial wrinkles, skin light aging, chloasma, pigmented nevus, acne scar, hypertrophic scar, vascular diseases, and so on.^[17–21]^

With the improvement of life, people pay more and more attention to external image, and pursue smooth and delicate skin. Clinicians and related scholars continue to invent and try various means to treat the skin lesions. The role of lattice laser has also been greatly affirmed. However, most of the research is the application of lattice laser combined with other treatments in beauty, but the research of purely lattice laser is less. This systematic evaluation and meta-analysis can provide evidence-based evidence for clinicians to use lattice laser in the treatment of facial rejuvenation. However, the study has some limitations. Due to the different ways of lattice laser in different studies, it has certain clinical heterogeneity and may affect the results to some extent. In addition, we only search for articles in Chinese and English, which may cause certain publication bias.

## Author contributions

**Data collection:** Dan Yan and Zechun Huang.

**Funding support:** Dan Yan.

**Literature retrieval:** Anli Zhang.

**Software operating:** Shuaihua Li.

**Supervision:** Yao Xiao.

**Writing – original draft:** Dan Yan and Zechun Huang.

**Writing – review & editing:** Dan Yan and Zechun Huang.
